# Panel-Specific Optimization and Benchmarking of Copy-Number Callers for ERBB2 Amplification Detection Using the Nanodigmbio NanOnco Plus Panel v3.0

**DOI:** 10.3390/diagnostics16203348

**Published:** 2026-10-14

**Authors:** Alexey Aleksandrovich Traspov, Peter Alekseevich Shatalov, Maria Alexandrovna Revkova, Polina Vladimirovna Mishakova, Elena Alekseevna Kirillova, Maria Pavlovna Raygorodskaya, Daria Valerievna Bagdasarova, Irina Alexeevna Leukhina, Victoria Sergeevna Surkova, Galina Fedorovna Mikhailova, Daria Alekseevna Averinskaya, Anna Petrovna Shinkarkina, Alena Valerevna Murzaeva, Andrei Dmitrievich Kaprin, Petr Viktorovich Shegai

**Affiliations:** 1 National Medical Research Radiological Centre of the Ministry of Health of the Russian Federation, 249036 Obninsk, Russia; 2 Scientific and Educational Resource Center for Innovative Technologies of Immunophenotyping, Digital Spatial Profiling and Ultrastructural Analysis, Patrice Lumumba Peoples’ Friendship University of Russia (RUDN University), 117198 Moscow, Russia

**Keywords:** ERBB2, HER2, copy-number alteration, targeted next-generation sequencing, fluorescence in situ hybridization, analytical validation, tumor purity, molecular diagnostics

## Abstract

**Background/Objectives:** ERBB2 amplification is a clinically relevant breast-cancer biomarker, but CNV detection from targeted sequencing depends on assay design, normalization, tumor fraction, and decision rules. We evaluated panel-specific ERBB2 analysis configurations against a binary FISH reference, with emphasis on the distinction between development-set performance and internal validation. **Methods:** We retrospectively evaluated 126 FFPE breast-tumor specimens (59 FISH-positive and 67 FISH-negative; 123 recorded patient identifiers) sequenced using NanOnco Plus Panel v3.0 on DNBSEQ-G400. Seven tumor-only, panel-compatible CNV caller configurations were compared; no matched normal tissue was available, although panel-matched normal reference pools were used where required by a caller. PureCN was run as a standalone tumor-only workflow, and CNVkit was run independently. Original fixed-rule results were calculated on the development cohort. An additional common 5-fold, 20-repeat patient-grouped cross-validation procedure selected decision rules using training data only, maximizing sensitivity subject to training specificity ≥90%. Upstream caller outputs were held fixed. Technical no-calls were retained. **Results:** On the development cohort, the fixed CNVkit median-bin log2 rule yielded 76.3% sensitivity (Wilson 95% CI, 64.0–85.3%) and 91.0% specificity (81.8–95.8%), with F1 81.8% and κ = 0.679. Native standalone PureCN yielded 16.9% sensitivity and 95.5% specificity at absCN ≥ 14; PURPLE had three numerical no-calls. Under the common patient-grouped decision-calibration procedure, CNVkit yielded 67.8% sensitivity and 91.0% specificity, whereas native PureCN yielded 19.3% sensitivity and 91.2% specificity. Exploratory computational purity subgroups and descriptive profiles across 554 genes illustrated heterogeneity of the measured DNA copy-number signal; independent pathology cellularity was unavailable, while quantitative FISH fields were recovered only for 45 of 126 specimens and were not used to define the full-cohort benchmark. **Conclusions:** The results support assay-specific development of ERBB2 decision rules but do not establish an intrinsic ranking of CNV software or clinical validation of other panel genes. The original full-cohort metrics are development-set estimates, and the additional cross-validation is conditional on previously generated caller outputs. External validation with independently assessed tumor content and complete clinical HER2 adjudication remains necessary.

## 1. Introduction

The current standard for determining HER2 status in breast cancer is based on immunohistochemistry (IHC) and, when required, in situ hybridization (ISH). IHC 3+ is interpreted as HER2-positive and IHC 0/1+ as HER2-negative, and IHC 2+ requires ISH assessment within the current ASCO/CAP testing framework [1]. Targeted next-generation sequencing (NGS) provides a complementary approach because copy-number information can be obtained from the same assay used to identify sequence variants.

Targeted hybrid-capture NGS can provide ERBB2 copy-number estimates that correlate with conventional HER2 testing. However, discordance has been reported in borderline, heterogeneous, and lower-tumor-cellularity specimens, indicating that NGS-derived copy number should be interpreted in the context of assay design and the underlying amplification state [2,3].

Copy-number analysis from targeted sequencing is affected by target-specific capture efficiency, GC content, mappability, DNA fragmentation, library preparation, and batch effects. Tumor purity and ploidy further modify the amplitude of the observed copy-number signal. These limitations are particularly relevant for smaller panels, where segmentation and baseline estimation are supported by a limited genomic footprint. Consequently, assay-matched controls, explicit operating thresholds, and validation across relevant tumor fractions and copy-number states are important components of analytical validation [4,5].

The compared workflows span distinct analytical strategies: CNVkit for relative target/antitarget depth normalization and segmentation [6,7]; ClinCNV for cohort-based normalization and CNV segmentation [8,9]; standalone PureCN for tumor-only purity/ploidy and absolute-copy-number modeling [10,11]; and the AMBER–COBALT–PURPLE stack for allelic-balance and depth-based absolute-copy-number fitting [12,13]. CESAR adds segmentation with anchor recalibration [14,15], BayesCNV hierarchical Bayesian copy-ratio estimation [16,17], and OFF-PEAK an on-/off-target reference and native CNV-event workflow [18,19]. We selected callers pragmatically to identify informative CNV signals in this assay without matched normal tissue while retaining distinct tumor-only and reference-pool strategies; the list is non-exhaustive and is not a systematic survey of all CNV callers. CESAR and BayesCNV were exploratory transfers from liquid-biopsy/amplicon settings; PURPLE was adapted from a predominantly whole-genome setting. ERBB2 was the diagnostic endpoint because binary orthogonal FISH labels were available for every included specimen. Other genes were examined descriptively without asserting diagnostic validation.

## 2. Materials and Methods

### 2.1. Study Design and Benchmark Cohort

This retrospective benchmark comprised 126 FFPE breast-tumor specimens with 59 FISH-positive and 67 FISH-negative results. As described in the original study protocol, eligibility required an interpretable binary ERBB2 FISH result and adequate targeted NGS data; specimens failing routine laboratory requirements for library construction, sequencing, processing, target coverage, or coverage uniformity, or lacking an interpretable FISH result, were excluded before caller comparison. These technical eligibility criteria were independent of ERBB2 amplification status. The complete screening log, numerical QC acceptance thresholds, and FISH-to-NGS block/section correspondence were not present in the retrieved analysis exports, so intentional enrichment and the disposition of screened cases remain unresolved. The cohort comprised 123 patients; four specimens from one patient represented repeat biopsies or DNA re-extractions. Resampling kept those four specimens together by patient identifier. Observed prevalence (46.8%) and prevalence-dependent metrics describe this cohort. Copy-number analysis used GRCh38 [20] and the shared ERBB2 analytical interval chr17:39,699,513–39,728,046 (zero-based start, end-exclusive overlap convention), which is an analytical window rather than the full gene locus.

The retrieved export does not preserve a complete screening log, enrichment history, or verified block/section linkage between FISH and NGS; these remain limitations of the retrospective reanalysis. FISH provenance comprised two source streams: our pathology team retained quantitative morphology/FISH fields for 45 specimens, whereas the transferred data for 81 specimens from an external collaborating organization contained binary FISH status without quantitative signal counts or group fields.

Panel-matched reference material comprised 176 blood-derived normal DNA samples. The archived configurations used this pool for PureCN, COBALT/PURPLE, CESAR, and BayesCNV; CNVkit used 167 normals that passed its reference QC. These distinct reference constructions were retained and were not rebuilt within cross-validation.

This was a retrospective study using previously obtained biological material. The study was conducted in accordance with the Declaration of Helsinki and approved by the Independent Ethics Council of the National Medical Research Radiological Centre of the Ministry of Health of the Russian Federation (protocol code: No. 138, approval date: 19 June 2026). Written informed consent for the research use of clinical and molecular data was obtained from the individuals who provided specimens for both groups.

### 2.2. Dna Extraction, Library Preparation, Target Capture, and Sequencing

Tumor DNA was extracted from FFPE sections using the QIAamp DNA FFPE Tissue Kit (QIAGEN, Hilden, Germany; cat. no. 56404) according to the manufacturer’s instructions [21]. DNA from peripheral-blood normal controls was extracted using the GM Blood M kit (Raissol/Sesana, Moscow, Russia) [22].

Libraries were constructed using the VAHTS Universal Plus DNA Library Prep Kit for MGI V2 (Vazyme, Nanjing, China; NDM627), with enzymatic fragmentation, and enriched using the NanOnco Plus Panel v3.0 MGI-compatible hybrid-capture workflow [23,24]. Sequencing used the DNBSEQ-G400 platform with PE150 reads [25]. Reagents, source versions, and configuration details are listed in Supplementary Table S3.

The aggregate laboratory QC summary was reported as descriptive assay context rather than a benchmark subgroup. Its median sample-level mean target coverage was 441.8× (range, 184.2–659.1×), and its median target coverage was 417.5× (range, 182–631×); further metrics are retained in Supplementary Table S7. The underlying technical export contains 36 records, of which 12 sample identifiers overlap the benchmark. A separate laboratory register linked by sample identifier to all 126 specimens has a median mean target coverage of 446.5× (range, 6.0–2164.1×) and a median target coverage of 419× (range, 6–1899×). Exact run/BAM correspondence for this register has not been established; these values are contextual and do not establish the QC of the original caller inputs or prove that study eligibility criteria were violated. CNVkit ERBB2 bin-depth summaries were available from score-compatible CNR files for 123 specimens.

### 2.3. Erbb2 Fish Reference Testing

ERBB2 FISH testing used the Wuhan HealthCare Biotechnology (Wuhan, China) HER2/CEP17 kit (REF FP-001) [26], with orange/red HER2 and green CEP17 probes. FFPE tissue sections underwent deparaffinization, pretreatment, denaturation, probe hybridization, and post-hybridization washing according to the manufacturer’s protocol.

Slides were examined with an AxioImager A-2 fluorescence microscope (Carl Zeiss Microscopy GmbH, Jena, Germany). DAPI counterstaining identified nuclei, and HER2/CEP17 signals were assessed in evaluable tumor regions. As specified in the original protocol description, only regions containing at least 10% identifiable tumor cells were scored, with approximately 20–60 evaluable tumor nuclei assessed per specimen to calculate the mean HER2 copies per cell and the HER2/CEP17 ratio. The 10% criterion is a region-selection criterion and is not a specimen-level percentage estimate of tumor cellularity. The benchmark comprised two FISH data streams: in 45/126 specimens, our pathology team retained quantitative HER2, CEP17, HER2/CEP17, and ISH-group fields from its morphology/FISH assessment; in 81/126 specimens, the transferred record from an external collaborating organization contained binary FISH status only, without quantitative signal counts or group fields.

For the 45 specimens assessed by our pathology team, the authors confirm that the FISH assessment was performed independently of the NGS analysis: readers were not given NGS caller outputs, PureCN purity estimates, or additional computational cellularity information. The recovered export does not retain the number or qualifications of these readers, whether scoring was independent at the individual-reader level, or how discrepant or borderline cases were resolved. For the 81 binary FISH results received from the external collaborating organization, reader qualifications, blinding documentation, and discrepancy-resolution records were not transferred to the study export. We therefore report the confirmed blinding of our 45-case assessment, identify the remaining reader-level metadata as unavailable, and do not infer reader counts or external-laboratory procedures.

The operational binary laboratory rule was positive for a HER2/CEP17 ratio ≥2.0 with mean HER2 copies ≥4.0 or a ratio <2.0 with mean HER2 copies ≥6.0. This is not the complete integrated ASCO/CAP IHC/ISH classification [1]: Groups 2–4 require IHC/ISH integration for final clinical interpretation. Quantitative FISH fields were present in 45 of 126 included specimens; the remaining 81 specimens had no quantitative HER2/CEP17 fields in the available export. The binary FISH status was retained for all 126 specimens as the benchmark reference. The occurrence and disposition of Groups 2–4 in the screened population cannot therefore be reconstructed, and concordance with the recorded binary reference must not be equated with complete clinical HER2-status concordance.

In the 45 records with quantitative fields, the recorded group field was Group 1 in 13, Group 2 in 2, Group 3 in 8, Group 4 in 5, and Group 5 in 17. These counts describe only the recovered subset. IHC results and final integrated laboratory reports were not present in the export, so no complete clinical reclassification is made.

As an exploratory source-consistency check limited to the 45 quantitative records, the recorded HER2/CEP17 ratio alone agreed with the recorded binary status in 33/45 specimens (73.3%; Cohen κ = 0.438), whereas the operational rule combining the ratio with the mean HER2 copies per cell agreed in 43/45 specimens (95.6%; Cohen κ = 0.910). Two records labeled binary-negative satisfy the recorded operational rule and remain flagged for verification against the primary scoring sheet and final laboratory report. One displayed ratio is not reproducible from its displayed mean HER2 and CEP17 values and is likewise flagged for source verification. These calculations are not a reclassification of the full cohort and do not provide a percentage estimate of tumor cellularity.

### 2.4. Sequence Preprocessing and Common Bam Input

FASTQ files were preprocessed with fastp 0.23.x [27] for adapter trimming, quality filtering, and pre-/post-processing quality summaries. Reads were aligned to the GRCh38 reference genome with BWA-MEM2 2.2.1 [28,29]. Duplicate templates were marked with streammd 4.3.0 [30], after which BAM files were coordinate-sorted and indexed with Sambamba 1.0.1 [31].

Base-quality score recalibration was performed with GATK 4.6.2.0 using BaseRecalibratorSpark and ApplyBQSRSpark [32,33]. The known-sites resources were dbSNP 146 [34], 1000 Genomes Phase 1 high-confidence SNVs [35], and Mills/1000 Genomes gold-standard indels [36]. The recalibrated BAM files were used as the common upstream input for all seven CNV methods.

### 2.5. Tumor Purity Assessment

The original study protocol describes morphological assessment of tumor cellularity on corresponding histologic material. For 45 specimens, our pathology team retained quantitative morphology/FISH fields, but no case-linked whole-specimen percentage cellularity values were available in the retrieved export. For the remaining 81 specimens, the transferred dataset contained only binary FISH status from the external collaborating organization. Computational purity estimates from native PureCN sample summaries were available for all 126 specimens [10,11] and were used only for exploratory stratification. The laboratory-register field labeled purity matched the native computational series in 126/126 specimens and could not be established as an independent morphology assessment. Consequently, a pathology–computational agreement analysis was not performed, and qualitative agreement is not asserted without paired percentage measurements.

In the 45-record quantitative subset, the HER2/CEP17 ratio was not associated with native PureCN computational purity (Spearman ρ = 0.034, *p* = 0.825). This is an exploratory source check rather than a pathology cellularity agreement analysis; missing quantitative FISH and pathology cellularity fields were not imputed.

Computational purity analyses were retained solely as exploratory, method-dependent descriptions at cumulative thresholds ≥10% to ≥90% and at a separate strict >70% threshold. They are not the requested independent pathology-based stratification. The intended PureCN purity/ploidy search configuration used a ploidy range of 1.4–5.0, but 96 of 126 archived reported ploidies fell outside that interval, indicating incorrect solutions or execution/provenance problems in the archived PureCN run. These values were not silently substituted with later model outputs; the discrepancy limits biological interpretation of the purity strata and absolute-copy-number estimates.

### 2.6. Analysis Configurations and Original Operating Points

CNVkit v0.9.14 [6,7] used the 167-normal pooled reference, MAPQ 10, and CBS with --threshold 0.0001, --drop-outliers 10, and --drop-low-coverage. The primary ERBB2 score was median target-bin log2. The archived fast_fish_validate.py procedure used stratified 5-fold cross-validation repeated three times (random seed 123), a grid of 51 training-score quantiles, and maximum training sensitivity subject to specificity ≥0.90, with MCC and then higher threshold as tie-breakers. The median of the 15 selected thresholds was 0.1587865; the strict comparator was 0.6465385. The original configurations are summarized in Table 1. Applying the single median threshold to the same 126 specimens produced Table 2, not out-of-fold predictions. Feature/configuration exploration was not nested within that historical validation.

ClinCNV v1.19.1 [8,9] was run with R 4.3.3 and Python 3.11; ngs-bits 2026_06 [37] was used for coverage-related utilities. The caller was evaluated in tumor-only mode with off-target coverage enabled. The primary profile used scoreS/lengthS 50/1, scoreG/lengthG 50/2, MAPQ 5, and an ERBB2-overlap requirement with CN_change ≥ 2. The internal ClinCNV minimumPurity parameter was set to 20; this is a software parameter and not a study-level tumor purity inclusion criterion.

PureCN [10,11] was run as a standalone tumor-only caller using its panel-matched NormalDB and native purity/ploidy and absolute-copy-number outputs. The revised benchmark uses the native PureCN ERBB2 output directly; CNVkit median-bin log2 values are evaluated independently as a separate workflow. A source-column error in the archived summary was corrected by extracting the ERBB2 C field directly from native PureCN gene output for all 126 specimens. The reported PureCN row was recomputed at the retained absCN ≥ 14 comparator; this retained threshold is not claimed to be an independently optimized native PureCN operating point. Thresholds ≥6, ≥8, ≥10, and ≥17 were recomputed as alternatives. The intended final ploidy search range was 1.4–5.0, but archived outputs included 96/126 reported ploidies outside that interval, consistent with incorrect solutions or a run-configuration problem. We retain those outputs transparently and do not claim that the observed out-of-range values represent valid biological ploidy estimates.

The tumor-only AMBER 4.2–COBALT 2.2–PURPLE 4.3 workflow was run using COBALT normalization with 176 normal BAMs and HMF GRCh38 pipeline resources version 2.3.0 [12,13]. The primary ERBB2 amplification rule was minCN > 3× sample ploidy, consistent with the high-level amplification framework used by the Hartwig Medical Foundation. PURPLE-derived purity and ploidy were not analyzed as study outcomes; ploidy was used only within the operational amplification rule.

CESAR [14,15] was run with the public CesaR repository, for which no tagged release was available, in the R analysis environment. The workflow used a 176-normal panel reference, a min_mean_depth of 200, and an anchor range of 80 to 100 with exclude_same_gene = TRUE. CR ≥ 1.1 was evaluated as an exploratory same-cohort operating point, while the CR ≥ 1.5 threshold was retained as a conservative comparator. Given that CESAR was developed for ctDNA and liquid biopsy, its use with FFPE hybrid-capture data was considered an exploratory domain adaptation. Archived CESAR commit: e36e6be169d4838b42d2401af373173b04dd7992.

BayesCNV [16,17] was run with the public repository, for which no tagged release was available, in Python 3.11.15. Target coverage was determined using mosdepth 0.3.14 [38], and the normal reference was the median coverage profile of the 176 normal samples. The CNV2Hierarchical ERBB2 model was assessed at μ ≥ 1.0, while μ ≥ 1.2 and μ ≥ 1.5 were examined as alternative operating points. Given that the original BayesCNV study utilized targeted amplicon cell-free DNA, this FFPE hybrid-capture application was considered exploratory. Archived BayesCNV commit: e6f247f81ff511d7049f43ec6bcc0256977a288b.

OFF-PEAK [18,19] was run from the public repository in the R analysis environment; no tagged software release was available. The unmodified 03_OFF-PEAK.R workflow was executed on a 302-BAM matrix containing 126 tumors and 176 normal controls. Parameters were mincor = 0.9, LOO = 1, downsample = 20,000, nbFake = 500, and minZ = 4. The native ERBB2-overlapping duplication call was assessed without ERBB2-specific retuning. Archived OFF-PEAK commit: 5c2b1050797c0c4cc87fdb393f76d0fd0abbeca6.

### 2.7. Statistical Analysis and Internal Decision Calibration

Confusion matrices and sensitivity, specificity, PPV, NPV, F1, accuracy, and Cohen’s κ [39] were recomputed from individual archived calls. Technical no-calls were excluded from each workflow-specific confusion matrix and reported separately; they were neither imputed nor used to inflate denominators. Table 2 reports development-set results for the original fixed rules, with specimen-level Wilson 95% CIs for sensitivity and specificity. The Wilson intervals do not account for repeated specimens from the same recorded patient. PPV, NPV, accuracy, F1, and κ depend on the selected cohort composition. Reanalysis was performed with the version-pinned Python environment supplied with the source package.

For the original fixed configurations, exact two-sided McNemar tests compare correctness on common evaluable specimens (Supplementary Table S5). These are exploratory descriptive tests: the rules were developed differently, multiplicity was not adjusted, and the original specimen-level tests do not account for the repeated patient identifier. Nominal *p*-values were not used to establish software superiority.

The additional common decision-calibration analysis used 5-fold StratifiedGroupKFold splits repeated 20 times, with seed 20260922 plus the zero-based repeat index, grouping by recorded patient identifier. The same splits were used for all configurations. On each training set, we selected a numeric threshold maximizing sensitivity subject to specificity ≥0.90; ties favored higher specificity and then higher threshold. Threshold candidates comprised unique observed training values plus an all-negative endpoint. Scores were CNVkit median-bin log2, native PureCN absCN, PURPLE minCN/ploidy, CESAR CR, and BayesCNV μ. For ClinCNV, the same objective selected among all 28 archived scoreS × lengthS profiles at CN_change ≥ 2, with an all-negative fallback; tied profiles were resolved deterministically by profile name. OFF-PEAK remained a fixed native comparator because a calibrated continuous score for every specimen was unavailable. Missing numerical outputs remained no-calls. Each specimen received one held-out prediction per repeat. Point estimates were calculated from confusion-matrix contributions averaged over repeats. Percentile 95% intervals used 2000 patient-cluster bootstrap resamples of these held-out contributions, keeping repeats together; these intervals condition on the fitted predictions and do not include complete pipeline-refitting uncertainty. Normalization, reference construction, upstream model fitting, and the original choice of score were not repeated within folds. Thus, this standardizes decision calibration on archived outputs, not end-to-end or external validation.

PureCN-derived computational purity defined exploratory cumulative strata (≥10% through ≥90% in 10-percentage-point steps), with a separate strict >70% subgroup. The first non-redundant stratum was ≥20%; the ≥60% and ≥70% strata are presented in the Results, and the full cumulative series is retained in Supplementary Table S1. Strata are nested and method-dependent and were selected descriptively; no independent pathology-based purity series was available. Additional methods, tables, and figures are available in the Supplementary Materials.

### 2.8. Use of Generative AI in Manuscript Preparation

Generative AI tools, including ChatGPT (OpenAI; GPT-6 was used for the final editorial review), were utilized to streamline drafting, language editing, harmonization of semantics, and generation of statistics and figures from data provided by the authors. The authors verified and interpreted the data. The authors confirm responsibility for the manuscript.

## 3. Results

### 3.1. Overall Caller Performance

**Figure 1:**
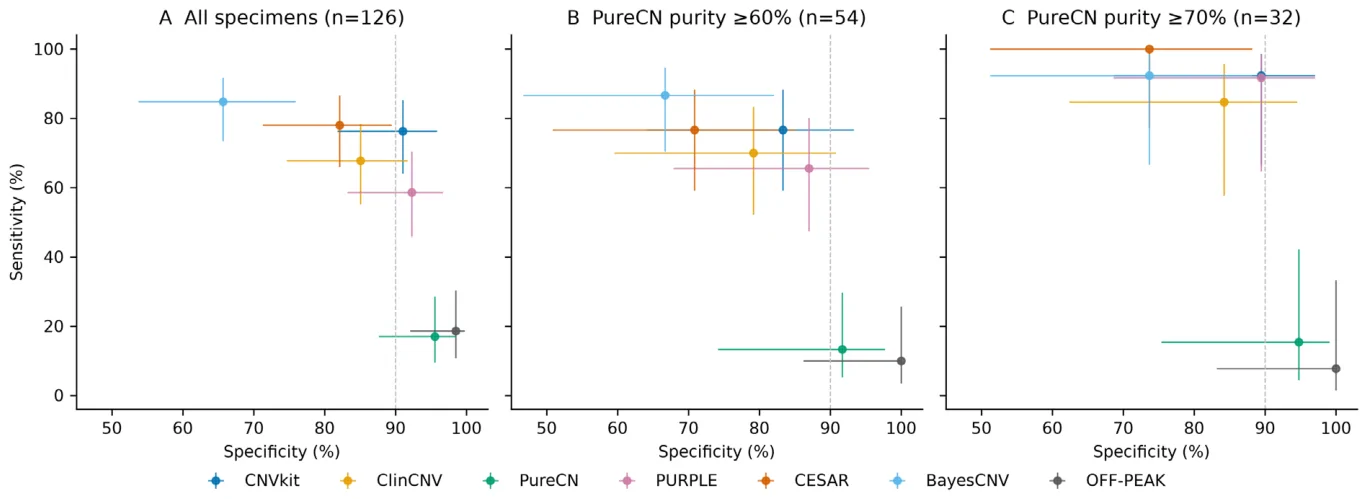
Consolidated performance at the retained fixed decision rules: (**A**) all 126 specimens; (**B**) computational purity ≥60% (*n* = 54); (**C**) computational purity ≥70% (*n* = 32). Horizontal and vertical error bars show Wilson 95% CIs for specificity and sensitivity, respectively. Panels (**B**,**C**) are exploratory, nested, PureCN-dependent subgroup descriptions; neither the subgroup thresholds nor the performance changes define a validated limit of detection. All three panels use development-set rules. Native PureCN sample-summary purity defines the strata. Vertical dashed reference lines indicate 90% specificity.

At the original fixed rules, CNVkit had the highest observed F1 (81.8%) and κ (0.679), with a sensitivity of 76.3% and a specificity of 91.0% on the development cohort. This is a comparison of differently configured workflows and does not establish intrinsic software superiority. CESAR yielded 78.0% sensitivity and 82.1% specificity; BayesCNV yielded 84.7% and 65.7%. Table 2 includes PPV, NPV, accuracy, and no-call counts; complete confusion matrices are provided in Supplementary Table S8. The consolidated whole-cohort and exploratory purity displays are shown in Figure 1.

ClinCNV yielded sensitivity/specificity of 67.8%/85.1%, native PureCN 16.9%/95.5% at absCN ≥ 14, and PURPLE 58.6%/92.3%. OFF-PEAK yielded 18.6%/98.5% under its native duplication rule. These differences combine score definition, configuration, and decision threshold with sample characteristics.

Native PureCN produced numerical outputs for all 126 specimens; all native run summaries had Flagged = TRUE and Failed = FALSE, while ERBB2 C.flagged was FALSE for all specimens. These quality indicators are distinct from numerical no-calls. PURPLE had three numerical no-calls (2.38%) and status NO_TUMOR in 102/126 specimens. Available numerical outputs were retained irrespective of these quality flags, limiting interpretation as clinically reportable results.

### 3.2. Alternative Operating Points

**Figure 2:**
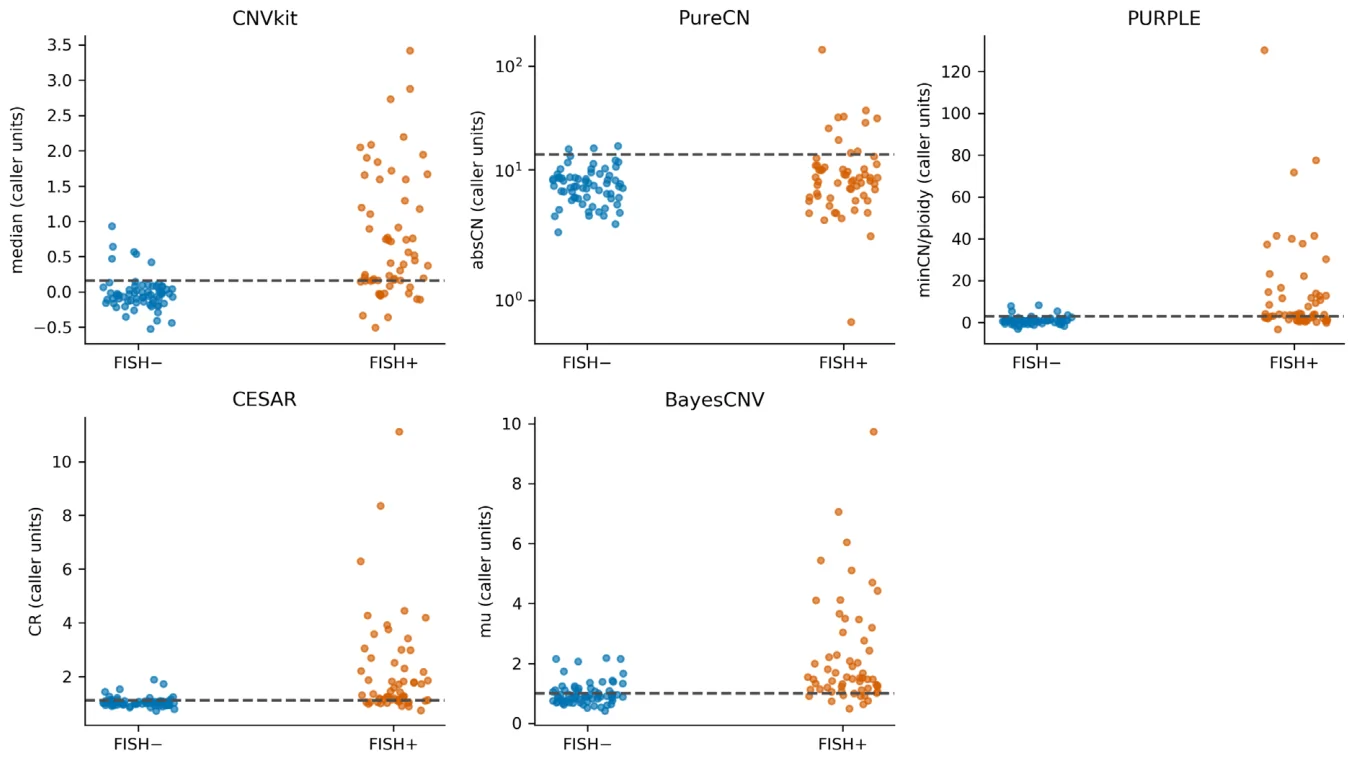
Continuous outputs by binary FISH status at the original fixed rules. Each point is one evaluable specimen; dashed lines show the original thresholds. Scores use different scales and are not interchangeable measures of gene copy number. The native PureCN absolute-CN panel uses a symmetric logarithmic axis to display extreme values. ClinCNV and OFF-PEAK were retained as profile-based and native-event comparisons. Dashed lines indicate the original fixed rules. ClinCNV is represented by a profile-based binary call and OFF-PEAK by its native duplication call; these comparators do not have a continuous score plotted. Scores use different scales, and the panels show development-set outputs. Blue points indicate FISH-negative specimens; orange points indicate FISH-positive specimens.

Alternative operating points changed the sensitivity–specificity tradeoff substantially (Supplementary Table S2; Figure 2). For native PureCN, lowering the absolute-copy-number threshold from ≥14 to ≥6 increased sensitivity from 16.9% to 69.5% while reducing specificity from 95.5% to 32.8%. Increasing CESAR CR from 1.1 to 1.5 reduced sensitivity from 78.0% to 45.8% while increasing specificity from 82.1% to 95.5%. For BayesCNV, increasing μ from 1.0 to 1.5 changed sensitivity/specificity from 84.7%/65.7% to 49.2%/91.0%. These within-workflow changes demonstrate the influence of decision rules without identifying a unique cause for between-workflow differences.

### 3.3. Exploratory Computational Purity Dependence

No independent pathology-based purity analysis could be performed. The native PureCN sample-summary purity results are provided for transparency in Supplementary Table S1. Across cumulative strata, sensitivity and specificity did not improve monotonically. At ≥70%, sensitivity was 92.3% for CNVkit, 84.6% for ClinCNV, 91.7% for PURPLE, and 100% for CESAR; only 32 specimens remained, compared with 54 at ≥60%. Specificity did not improve uniformly. The subgroups differ in composition and are defined by a computational model used within one benchmark configuration, precluding causal attribution to tumor fraction.

The ≥80% and ≥90% strata contained only 19 and six specimens. All purity subgroup results are exploratory. The ≥60% and ≥70% displays are combined with the whole-cohort display in Figure 1; the full cumulative analysis and exact denominators remain in Supplementary Tables S1 and S9.

### 3.4. Exploratory Strict High-Purity and Paired Comparisons

In the separate strict >70% high-purity subgroup (*n* = 30; 13 FISH-positive and 17 FISH-negative tumors), CNVkit achieved 92.3% sensitivity and 94.1% specificity. PURPLE achieved 91.7% sensitivity and 94.1% specificity among 29 evaluable tumors, while CESAR detected all 13 FISH-positive tumors but classified three FISH-negative tumors as positive. Detailed subgroup metrics are provided in Supplementary Table S4.

Exploratory specimen-level McNemar comparisons are retained in Supplementary Table S5 to document the original fixed-rule results. Unequal rule development, correlated specimens, and unadjusted multiple comparisons prevent interpreting these nominal *p*-values as confirmatory evidence of algorithmic superiority.

### 3.5. Common Internal Decision Calibration

The common patient-grouped resampling procedure yielded sensitivity/specificity of 67.8%/91.0% for CNVkit, 59.5%/90.4% for ClinCNV, 19.3%/91.2% for native PureCN, 63.6%/89.2% for PURPLE, 60.5%/89.9% for CESAR, and 51.9%/90.2% for BayesCNV (Table 3). OFF-PEAK remained at 18.6%/98.5% because its rule was fixed. The training specificity constraint does not guarantee ≥90% specificity on held-out specimens. These conditional internal estimates are distinct from external validation.

An exact rerun of the original CNVkit script reproduced its median threshold (0.1587865), fixed-rule counts (45 TP, 6 FP, 14 FN, 61 TN), and historical 5-fold ×3 pooled held-out sensitivity 72.9% and specificity 91.0% (129 TP, 48 FN, 183 TN, 18 FP across 378 repeated predictions). Those 378 predictions represent 126 specimens evaluated three times, not 378 independent specimens. The historical feature/configuration choice was non-nested. Its threshold grid, MCC tie-breaker, and specimen-level split design differ from the new common patient-grouped procedure.

### 3.6. Discordant Cases and Multi-Gene Signal Context

At least one available configuration disagreed with binary FISH in 87 of 126 specimens. The case-level supplement reports all seven outputs, fixed-rule calls, discordance classes, computational purity, available coverage summaries, and explicit missingness for clinical fields. CNVkit had 14 false negatives and six false positives. Their median log2 scores were −0.037 and 0.555, respectively, compared with −0.061 in true negatives and 0.751 in true positives. The corresponding median ERBB2 bin depths were approximately 494×, 706×, 497×, and 1074× in score-compatible files. Thus, discordance was not uniformly characterized by absent sequencing coverage; depth alone cannot establish the biological amplification state.

**Figure 3:**
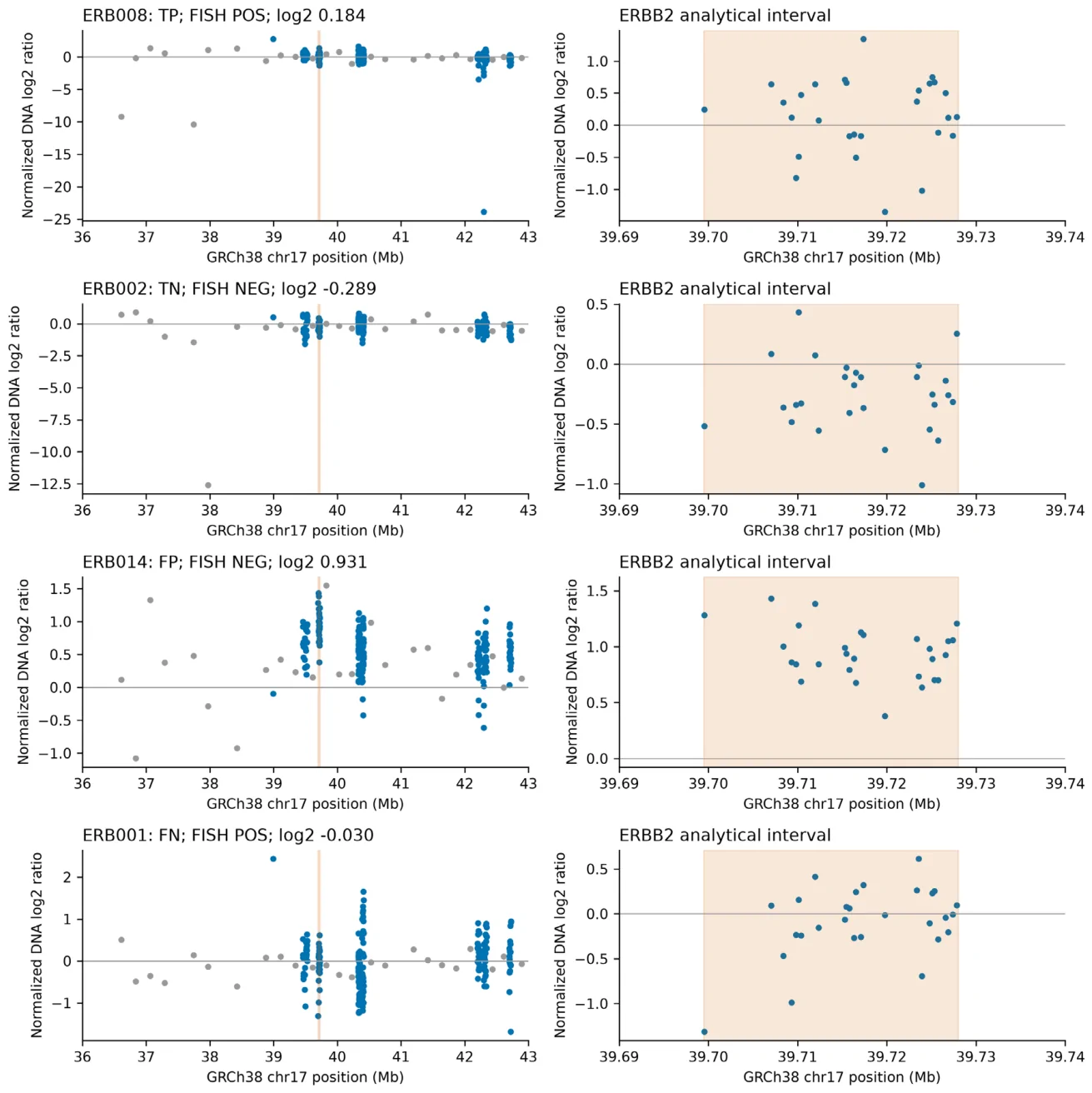
CNVkit DNA-depth profiles for deterministic examples of TP, TN, FP, and FN outcomes under the original rule. (**Left**): chr17 regional context; (**right**): the ERBB2 analytical window. Blue points denote target bins, and gray points antitarget bins; the shaded interval marks the analytical ERBB2 window. Positions use GRCh38. Panels display normalized DNA read depth, not RNA expression or FISH signal counts.

Available CNR files reproduced the archived median ERBB2 log2 score within 10^−4^ for 123/126 specimens. Three mismatching current files were excluded from regional and multi-gene analyses, without changing the original binary metrics. Compatible profiles contained 28 ERBB2-overlapping bins. Figure 3 shows one deterministic example per CNVkit outcome (the first pseudonymous case identifier in each category), avoiding selection for visually striking patterns. Across these 123 specimens, 554 gene annotations had at least three bins in every specimen. Supplementary Table S12 and Figure S1 describe within-gene bin MAD and between-specimen variation. These are descriptive DNA-depth signals; neither additional experimental confirmation nor a panel-wide diagnostic threshold was available.

## 4. Discussion

This study evaluates ERBB2 analysis configurations on a single targeted hybrid-capture assay. Relative-depth scores, purity/ploidy-adjusted absolute-CN estimates, and native-event calls measure different quantities and use different decision procedures [4,5]. PureCN was evaluated as a standalone tumor-only workflow, whereas the CNVkit benchmark used its independent relative-depth output.

CNVkit had the highest observed F1 among the original fixed configurations, but Table 2 is a development-set comparison. The historical 5-fold, 3-repeat CNVkit procedure and the new common patient-grouped procedure address different calibration questions and yield different sensitivity estimates. The latter applies a uniform specificity objective and separates decision fitting from held-out predictions while leaving upstream outputs and previous configuration decisions fixed. Its estimates support further evaluation of CNVkit for this panel but not a definitive comparative ranking of software.

The standalone PureCN comparison illustrates how purity, ploidy, absolute-copy-number modeling, and operating threshold jointly determine a positive call. Its high-specificity absCN threshold sacrifices sensitivity; lowering it produces substantially more positive calls. The archived out-of-range ploidy values indicate execution or model-solution problems and limit biological interpretation; they should not be generalized to the PureCN algorithm itself.

PURPLE was evaluated using a high-level, ploidy-scaled rule in a targeted tumor-only adaptation. Its frequent NO_TUMOR status is an additional reason to distinguish a numerical comparison from a validated clinical reporting workflow. Missing numerical outputs and quality-status flags have different meanings and must both be disclosed. The observed high-purity subgroup performance cannot resolve whether fitting, score calibration, or sample composition explains the apparent improvement.

CESAR and BayesCNV extend the possible range of operating scenarios but differ in their original assay contexts from the present study. CESAR was developed for CNV detection in ctDNA/liquid-biopsy data [14], and BayesCNV was developed for copy-number estimation in targeted amplicon cell-free DNA [16]. Consequently, the use of both tools on FFPE hybrid-capture data should be considered as exploratory domain adaptation and would require additional validation to set clinical operating thresholds.

OFF-PEAK exhibited the opposite behavior, with high specificity and low ERBB2 sensitivity under the native duplication call. OFF-PEAK was designed to utilize on-target, off-target, and noncoding reads for broader CNV detection [18,19]. Because no ERBB2-specific retuning was performed, its result in this benchmark should be interpreted as a conservative native-software comparator rather than as a comprehensive assessment of the method.

Per-case outputs provide a more direct account of discordance than binary summary metrics alone. CNVkit false negatives had median ERBB2 log2 −0.037, similar to −0.061 among true negatives, whereas false positives had median 0.555 and true positives 0.751. Raw-bin profiles show the available depth signal and its regional context. These observations do not identify the biological cause: without full-cohort quantitative FISH, independently assessed cellularity, and verified tissue matching, low-level amplification, heterogeneity, sampling differences, and assay/normalization effects remain unresolved possibilities. DNA read-depth plots are not expression measurements.

Published limits of detection for CNV calling should not be interpreted as universal tumor purity thresholds because they depend on alteration magnitude, assay design, normalization, and the selected operating point. Ross et al. demonstrated this for ERBB2 using MSK-IMPACT: a moderately amplified cell line with a HER2/CEP17 ratio of 4.2 remained above the NGS amplification threshold at 12.5% tumor DNA, whereas a low-level amplified cell line with a HER2/CEP17 ratio of 2.4 exceeded the NGS amplification threshold only at 50% tumor DNA [2]. Therefore, FISH-positive tumors should not be considered a homogeneous copy-number class when analytical sensitivity is evaluated.

Assay-specific optimization can materially alter apparent CNV performance at low tumor fractions. Chandramohan et al. validated an optimized tumor-only CNVkit workflow with 100% sensitivity and 93% specificity in an independent cohort. In dilution studies, a high-level CCND1 amplification was detected at approximately 20% tumor purity, whereas copy-loss events required approximately 40–50% purity [4]. Xia et al. studied 294 breast cancers using a 275–300-gene hybrid-capture assay with 166 probes across the 26 ERBB2 exons. After exclusion of samples with <20% tumor cellularity, the optimal ERBB2 median log2-ratio cutoff achieved 84.3% sensitivity and 98.7% specificity [3]. Importantly, the 13 discordant cases had tumor cellularities of 35–100% (mean, 69%), and adjustment for tumor cellularity did not improve overall classification [3]. These findings support interpreting the ≥70% effect in our cohort as assay-specific rather than as a universal biological constraint of CNV detection.

Limitations include the retrospective selected cohort, unknown screening/enrichment history, one repeated patient identifier, clinical HER2 adjudication details not represented in the analysis export, unavailable case-linked percentage pathology cellularity, quantitative FISH fields retained for only 45/126 specimens with binary external FISH status for the remaining 81 specimens, reader-level metadata and blinding logs unavailable for the external results, unavailable tissue-matching documentation, and the source-provenance limitations described above. The original rules were developed asymmetrically, and the new resampling validates only decision calibration on previously generated outputs. Cohort-based normalization can depend on other specimens even without their labels, so this is not a prospectively isolated pipeline evaluation. Other-gene signal dispersion includes biological CNV variation and technical variation and is not a measurement of pure technical error or diagnostic accuracy. No additional gene-specific experimental validation was performed. Comparable peak-RAM and wall-time measurements were not recoverable for the historical runs, and no runtime ranking is reported. An instrumented benchmarking protocol is supplied with the source package for prospective use.

The main strengths are the use of the shared BAM-derived cohort, panel-matched reference material, a binary FISH label for each included specimen, explicit no-call handling, and reproducible case-level calculations. The revised source package includes decision-calibration code, fold assignments, score definitions, and figure-generation scripts. Independent external testing, with clinical adjudication and consistent upstream fitting, is still required.

## 5. Conclusions

For this panel and binary ERBB2 endpoint, the original CNVkit median-bin log2 ≥0.1587865 rule yielded development-set sensitivity of 76.3% and specificity of 91.0%. The common patient-grouped internal decision-calibration analysis yielded 67.8% sensitivity and 91.0% specificity for the fixed median-bin score. These estimates describe different assessment procedures and should not be interchanged.

Computational purity subgroup patterns and multi-gene depth profiles are exploratory. They neither validate a universal tumor purity cutoff nor establish diagnostic performance for other genes. Native PureCN results are conditional on the archived run configuration and its documented ploidy-solution limitations.

The results support panel-specific workflow development and motivate independent validation; they do not justify standalone clinical decision rules or a definitive ranking of the software packages.

## Figures and Tables

**Table 1:** Original panel-specific analysis configurations. Decision rules were developed asymmetrically; these configurations are not equivalently optimized native caller implementations. PureCN and CNVkit were run independently as standalone caller workflows.

Caller	Version	Primary ERBB2 Positive Rule	Threshold Status
CNVkit	0.9.14	Median ERBB2 target-bin log2 ≥ 0.1587865	Historical 5-fold ×3 CV; fixed threshold re-applied to development cohort
ClinCNV	v1.19.1	ERBB2 overlap + CN_change ≥ 2	Sensitive S50/L1 profile; non-nested grid tuning
PureCN	Standalone tumor-only model; run-version caveat in Table S3	Native ERBB2 absolute CN ≥ 14	Retained ≥14 comparator; native-output row recomputed after source correction
PURPLE	AMBER 4.2/COBALT 2.2/PURPLE 4.3	ERBB2 minCN > 3× sample ploidy	Ploidy-scaled high-level amplification rule
CESAR	Public repository; no tagged release	CR ≥ 1.1	Exploratory same-cohort cutoff
BayesCNV	Public repository; no tagged release	μ ≥ 1.0	Exploratory hybrid-capture adaptation
OFF-PEAK	Public repository; no tagged release	Native ERBB2-overlapping DUP	Native default comparator; no retuning

**Table 2:** Development-set performance at the retained fixed decision rules against binary FISH. The selected rules were applied back to the same cohort; these are not held-out validation estimates. Wilson 95% CIs are specimen-level. Evaluable denominators exclude technical no-calls. PPV, NPV, accuracy, F1, and κ reflect this selected cohort (59/126 FISH-positive). The PureCN row was recomputed from standalone native output after correcting the source-column attribution; its retained C ≥ 14 threshold was not independently optimized on native output.

Caller	Eval./NC	Sensitivity, % (95% CI)	Specificity, % (95% CI)	PPV, %	NPV, %	F1, %	Accuracy, %	κ
CNVkit	126/0	76.3 (64.0–85.3)	91.0 (81.8–95.8)	88.2	81.3	81.8	84.1	0.679
ClinCNV	126/0	67.8 (55.1–78.3)	85.1 (74.7–91.7)	80.0	75.0	73.4	77.0	0.534
PureCN	126/0	16.9 (9.5–28.5)	95.5 (87.6–98.5)	76.9	56.6	27.8	58.7	0.131
PURPLE	123/3	58.6 (45.8–70.4)	92.3 (83.2–96.7)	87.2	71.4	70.1	76.4	0.518
CESAR	126/0	78.0 (65.9–86.6)	82.1 (71.3–89.4)	79.3	80.9	78.6	80.2	0.601
BayesCNV	126/0	84.7 (73.5–91.8)	65.7 (53.7–75.9)	68.5	83.0	75.8	74.6	0.497
OFF-PEAK	126/0	18.6 (10.7–30.4)	98.5 (92.0–99.7)	91.7	57.9	31.0	61.1	0.180

**Table 3:** Internal validation of common decision calibration on archived outputs. Point estimates average held-out confusion contributions across 20 repeats; 95% intervals are percentile patient-cluster bootstrap intervals (2000 resamples) conditional on the fitted predictions. Upstream models and reference construction were not refitted within folds. OFF-PEAK is an untuned native comparator.

Caller	Eval./NC	Sensitivity, % (95% CI)	Specificity, % (95% CI)	F1, %	Accuracy, %
CNVkit	126/0	67.8 (57.3–77.4)	91.0 (83.3–97.3)	76.2	80.2
ClinCNV	126/0	59.5 (47.3–71.1)	90.4 (83.3–96.5)	69.9	76.0
PureCN	126/0	19.3 (10.2–29.5)	91.2 (83.9–96.5)	29.9	57.5
PURPLE	123/3	63.6 (51.8–74.8)	89.2 (80.9–96.0)	72.4	77.1
CESAR	126/0	60.5 (48.7–71.9)	89.9 (82.5–96.3)	70.4	76.2
BayesCNV	126/0	51.9 (40.0–63.3)	90.2 (82.8–96.4)	63.7	72.3
OFF-PEAK	126/0	18.6 (8.8–28.8)	98.5 (95.0–100.0)	31.0	61.1

## Data Availability

Human sequencing and clinical source records are not publicly available because of privacy and legal restrictions. The revision package contains analysis source code and a separately packaged pseudonymized case-level dataset; the latter remains subject to applicable institutional and consent requirements. No public repository accession has yet been assigned. Permitted de-identified data may be requested from the corresponding author.

## Supplementary Materials

The following supporting information can be downloaded at: https://www.mdpi.com/article/10.3390/diagnostics16203348/s1. Supplementary Materials include detailed processing and configuration methods; original and alternative decision rules; purity subgroup metrics; exact source revisions; no-call accounting; contextual and case-linked QC definitions; a common grouped-CV analysis specification; source-provenance checks; descriptive multi-gene variability; and pseudonymized case-level outputs and reproducibility code. Supplementary Methods S1. FASTQ-to-BAM Processing; Supplementary Methods S2. Detailed Caller Configurations; Supplementary Methods S3. No-Call Handling and Purity Stratification; Table S1. Sensitivity and specificity across cumulative PureCN-estimated tumor-purity strata. Each caller cell is sensitivity/specificity (%); Table S2. Alternative caller operating points calculated from raw per-sample calls; Table S3. Reagents, platforms, software versions, study functions, and official sources. Website sources were accessed on 20 August 2026; Table S4. Performance in the strict high-purity subgroup (>70%; n = 30; 13 FISH-positive and 17 FISH-negative). Sensitivity and specificity are shown with Wilson 95% confidence intervals; Table S5. Exact two-sided McNemar comparisons of caller correctness versus CNVkit on common evaluable samples; Table S6. Technical no-call summary. Sample identifiers are omitted from the publication table; Table S7. Contextual laboratory QC from a separate 36-record export (12 sample identifiers overlap the benchmark); Table S8. Complete primary diagnostic metrics against the FISH reference; Table S9. Sensitivity and specificity with Wilson 95% confidence intervals for the retained cumulative purity strata (≥20%, ≥60%, and ≥70%); Supplementary Methods S4. Common Decision Calibration; Supplementary Methods S5. Source Audit and Descriptive Profiles; Table S10. Scope of the common decision-calibration analysis; Table S11. CNVkit discordance summaries at the original fixed threshold; Table S12. Descriptive variation for selected panel genes in 123 specimens with compatible CNR files; Figure S1. Descriptive normalized DNA-depth variation for selected panel genes. The machine-readable case dataset preserves unavailable clinical fields as missing. Figure S2. Target-bin DNA-depth profiles at six other panel genes for the same four deterministic cases used in Figure 3.

## References

[1] Wolff A.C., Somerfield M.R., Dowsett M., Hammond M.E.H., Hayes D.F., McShane L.M., Saphner T.J., Spears P.A., Allison K.H. (2023). Human Epidermal Growth Factor Receptor 2 Testing in Breast Cancer. Arch. Pathol. Lab. Med..

[2] Ross D.S., Zehir A., Cheng D.T., Benayed R., Nafa K., Hechtman J.F., Janjigian Y.Y., Weigelt B., Razavi P., Hyman D.M. (2017). Next-Generation Assessment of Human Epidermal Growth Factor Receptor 2 (ERBB2) Amplification Status: Clinical Validation in the Context of a Hybrid Capture-Based, Comprehensive Solid Tumor Genomic Profiling Assay. J. Mol. Diagn..

[3] Xia D., Kuo F., Hughes M., Lindeman N., Manning D., Files J., Strauss S., Kirkner G., Mohammed-Abreu A., Winer E. (2024). HER2/ERBB2 Copy Number Analysis by Targeted Next-Generation Sequencing in Breast Cancer. Am. J. Clin. Pathol..

[4] Chandramohan R., Reuther J., Gandhi I., Voicu H., Alvarez K.R., Plon S.E., Lopez-Terrada D.H., Fisher K.E., Parsons D.W., Roy A. (2022). A Validation Framework for Somatic Copy Number Detection in Targeted Sequencing Panels. J. Mol. Diagn..

[5] Rieber N., Bohnert R., Ziehm U., Jansen G. (2017). Reliability of Algorithmic Somatic Copy Number Alteration Detection from Targeted Capture Data. Bioinformatics.

[6] Talevich E., Shain A.H., Botton T., Bastian B.C. (2016). CNVkit: Genome-Wide Copy Number Detection and Visualization from Targeted DNA Sequencing. PLoS Comput. Biol..

[7] CNVkit Project CNVkit Release v0.9.14. https://github.com/etal/cnvkit/releases/tag/v0.9.14.

[8] Demidov G., Ossowski S. (2019). ClinCNV: Novel Method for Allele-Specific Somatic Copy-Number Alterations Detection. bioRxiv.

[9] ClinCNV Project. Official Repository. https://github.com/imgag/ClinCNV.

[10] Riester M., Singh A.P., Brannon A.R., Yu K., Campbell C.D., Chiang D.Y., Morrissey M.P. (2016). PureCN: Copy Number Calling and SNV Classification Using Targeted Short Read Sequencing. Source Code Biol. Med..

[11] PureCN Project. Official Repository. https://github.com/lima1/PureCN.

[12] Priestley P., Baber J., Lolkema M.P., Steeghs N., de Bruijn E., Shale C., Duyvesteyn K., Haidari S., van Hoeck A., Onstenk W. (2019). Pan-Cancer Whole-Genome Analyses of Metastatic Solid Tumours. Nature.

[13] Hartwig Medical Foundation hmftools/PURPLE Repository. https://github.com/hartwigmedical/hmftools.

[14] Lu J., Ni S., Wang L., Wu N., Jiang X. (2026). CESAR: A R Package for High-Sensitivity Detection of Copy Number Variations in ctDNA Using Segmentation and Anchor Recalibration. bioRxiv.

[15] CESAR Project. Official Repository. https://github.com/nishuai/CesaR.

[16] Talbot A., Kotlar A., Rishishwar L., Conley A., Zhao M., Yang N., Liu M., Wang Z., Polvino S., Ke Y. (2026). BayesCNV: A Bayesian Hierarchical Model for Sensitive and Specific Copy Number Estimation in Cell Free DNA. Diagnostics.

[17] BayesCNV Project. Official Repository. https://github.com/Pillar-Biosciences-Inc/BayesCNV.

[18] Quinodoz M., Kaminska K., Cancellieri F., Han J.H., Peter V.G., Celik E., Janeschitz-Kriegl L., Schärer N., Hauenstein D., György B. (2024). Detection of Elusive DNA Copy-Number Variations in Hereditary Disease and Cancer through the Use of Noncoding and Off-Target Sequencing Reads. Am. J. Hum. Genet..

[19] OFF-PEAK Project. Official Repository. https://github.com/mquinodo/OFF-PEAK.

[20] Schneider V.A., Graves-Lindsay T., Howe K., Bouk N., Chen H.-C., Kitts P.A., Murphy T.D., Pruitt K.D., Thibaud-Nissen F., Albracht D. (2017). Evaluation of GRCh38 and de novo Haploid Genome Assemblies Demonstrates the Enduring Quality of the Reference Assembly. Genome Res..

[21] QIAGEN QIAamp DNA FFPE Tissue Kit, Cat. No. 56404. https://www.qiagen.com/fm/products/discovery-and-translational-research/dna-rna-purification/dna-purification/genomic-dna/qiaamp-dna-ffpe-tissue-kit.

[22] Sesana Raissol GM Blood M DNA Extraction Kit. https://products.sesana.ru/raissoleng.

[23] Vazyme Biotech VAHTS Universal Plus DNA Library Prep Kit for MGI V2, NDM627. https://www.vazymeglobal.com/product-center/dna-library-preparation-kit.

[24] Nanodigmbio NanOnco Plus Panel v3.0. https://www.nanodigmbio.com/Solid-Tumor/819.html.

[25] MGI Tech DNBSEQ-G400 Sequencing Platform and Resources. https://en.mgi-tech.com/Products/reagents/du_id/4/ping_id/1/category_id/.

[26] Wuhan HealthCare Biotechnology FISH Reagents: Human HER2 Gene Amplification Detection Kit, FP-001. https://healthcare-biotech.com/list_49.html.

[27] Chen S., Zhou Y., Chen Y., Gu J. (2018). fastp: An Ultra-Fast All-in-One FASTQ Preprocessor. Bioinformatics.

[28] Vasimuddin M., Misra S., Li H., Aluru S. Efficient Architecture-Aware Acceleration of BWA-MEM for Multicore Systems. Proceedings of the 2019 IEEE International Parallel and Distributed Processing Symposium (IPDPS).

[29] BWA-MEM2 Project BWA-MEM2 Release v2.2.1. https://github.com/bwa-mem2/bwa-mem2/releases/tag/v2.2.1.

[30] Leonard C. (2023). streammd: Fast Low-Memory Duplicate Marking Using a Bloom Filter. Bioinformatics.

[31] Tarasov A., Vilella A.J., Cuppen E., Nijman I.J., Prins P. (2015). Sambamba: Fast Processing of NGS Alignment Formats. Bioinformatics.

[32] Van der Auwera G.A., Carneiro M.O., Hartl C., Poplin R., del Angel G., Levy-Moonshine A., Jordan T., Shakir K., Roazen D., Thibault J. (2013). From FastQ Data to High-Confidence Variant Calls: The Genome Analysis Toolkit Best Practices Pipeline. Curr. Protoc. Bioinform..

[33] Broad Institute Genome Analysis Toolkit (GATK), Release 4.6.2.0. https://github.com/broadinstitute/gatk/releases/tag/4.6.2.0.

[34] Sherry S.T., Ward M.H., Kholodov M., Baker J., Phan L., Smigielski E.M., Sirotkin K. (2001). dbSNP: The NCBI Database of Genetic Variation. Nucleic Acids Res..

[35] 1000 Genomes Project Consortium (2015). A Global Reference for Human Genetic Variation. Nature.

[36] Mills R.E., Pittard W.S., Mullaney J.M., Farooq U., Creasy T.H., Mahurkar A.A., Kemeza D.M., Strassler D.S., Ponting C.P., Webber C. (2011). Natural Genetic Variation Caused by Small Insertions and Deletions in the Human Genome. Genome Res..

[37] ngs-bits Project. Official Repository. https://github.com/imgag/ngs-bits.

[38] Pedersen B.S., Quinlan A.R. (2018). Mosdepth: Quick Coverage Calculation for Genomes and Exomes. Bioinformatics.

[39] Cohen J. (1960). A Coefficient of Agreement for Nominal Scales. Educ. Psychol. Meas..

