# Supplementary material for: Panel-Specific Optimization and Benchmarking of Copy-Number Callers for ERBB2 Amplification Detection Using the Nanodigmbio NanOnco Plus Panel v3.0
Source: Diagnostics (Basel). 2026 Oct 14;16(20):3348. doi: 10.3390/diagnostics16203348 (PMC13703794; doi:10.3390/diagnostics16203348)
Supplement: Supplementary file 1 [file diagnostics-16-03348-s001.zip › diagnostics-4566699-supplementary.pdf]

Supplementary Materials

Panel-Specific Optimization and Benchmarking of Copy-Number Callers for ERBB2 Amplification Detection Using the Nanodigmbio NanOnco Plus Panel v3.0

Alexey Aleksandrovich Traspov[1], Peter Alekseevich Shatalov[1], Maria Alexandrovna Revkova[1], Polina Vladimirovna Mishakova[1], Elena Alekseevna Kirillova[1], Maria Pavlovna Raygorodskaya[1], Daria Valerievna Bagdasarova[1], Irina Alexeevna Leukhina[1], Victoria Sergeevna Surkova[1], Galina Fedorovna Mikhailova[1], Daria Alekseevna Averinskaya[1], Anna Petrovna Shinkarkina[1], Alena Valerevna Murzaeva[1], Andrei Dmitrievich Kaprin[1,2], Petr Viktorovich Shegai[1,2].

[1] National Medical Research Radiological Centre of the Ministry of Health of the Russian Federation, 249036 Obninsk, Russia

[2] Scientific and Educational Resource Center for Innovative Technologies of Immunophenotyping, Digital Spatial Profiling and Ultrastructural Analysis, Patrice Lumumba Peoples' Friendship University of Russia (RUDN University), 117198 Moscow, Russia

Correspondence: A.A. Traspov; traspovalex@gmail.com

Supplementary Methods S1. FASTQ-to-BAM Processing

Paired-end reads were processed with fastp 0.23.x for adapter trimming, quality filtering, and quality summaries [13]. Reads were aligned to GRCh38 with BWA-MEM2 2.2.1 [14,15]. Duplicate templates were marked with streammd 4.3.0 [16], and BAM files were coordinate-sorted and indexed with Sambamba 1.0.1 [17]. Base-quality score recalibration was performed with GATK 4.6.2.0 using BaseRecalibratorSpark and ApplyBQSRSpark [18,19], with dbSNP 146, 1000 Genomes Phase 1 high-confidence SNVs, and Mills/1000 Genomes gold-standard indels as known-sites resources [20–22].

Supplementary Methods S2. Detailed Caller Configurations

CNVkit used the original configuration described in Section 2.6. The threshold of 0.1587865 is the median from 15 training-fold thresholds in fast_fish_validate.py (5 folds ×3 repeats, seed 123; 51-quantile training grid; sensitivity maximization at specificity ≥0.90; ties by MCC then higher threshold). Table 2 in the main text applies this single threshold back to the development cohort. An exact rerun reproduced the original threshold, fixed full-cohort counts, and historical pooled held-out sensitivity 72.9% and specificity 91.0%; repeated observations must not be used as independent denominators for confidence intervals.

ClinCNV v1.19.1 was run with R 4.3.3 and Python 3.11. Coverage-related utilities were provided by ngs-bits 2026_06. The primary caller profile used scoreS/lengthS 50/1, scoreG/lengthG 50/2, MAPQ 5, off-target coverage, and an ERBB2-overlap requirement with CN_change ≥2. The internal minimumPurity parameter was 20. Baseline S100/L4 and strict S150/L1 profiles were retained as alternatives.

The archived native PureCN output came from a standalone tumor-only workflow with a 176-normal NormalDB. A later configuration snapshot records PureCN 2.18.0 (Bioconductor 3.23), beta-binomial allelic modeling, PSCBS segmentation, two eigenvectors, off-target fraction 1, purity search 0.05–0.95, and intended final ploidy search 1.4–5.0; the exact version and complete configuration of the output-producing run remain unverified. The revised benchmark uses native PureCN absolute-copy-number values directly, while CNVkit median-bin log2 values are evaluated independently. The ERBB2 C field from native gene output corrected a source-column error in the archived summary. All PureCN tables were recomputed at the retained absCN ≥14 comparator and alternatives ≥6, ≥8, ≥10, and ≥17. This retained threshold is not claimed to be an independently optimized native operating point. The archived output contains 96/126 ploidy values outside 1.4–

5.0, indicating incorrect solutions or an execution/provenance problem; these values are retained and flagged rather than silently replaced.

AMBER 4.2 (quay.io/biocontainers/hmftools-amber:4.2--hdfd78af_0), COBALT 2.2 (quay.io/biocontainers/hmftools-cobalt:2.2--hdfd78af_0), and PURPLE 4.3 (quay.io/biocontainers/hmftools-purple:4.3--hdfd78af_0) were used with hmf_pipeline_resources.38_v2.3.0--2. The workflow was run in tumor-only mode with 176-normal COBALT normalization and somatic VCF input disabled. The primary amplification rule was ERBB2 minCN >3× sample ploidy. The HMF driver AMP flag was evaluated as an alternative comparator.

CESAR was run from the public CesaR repository; no tagged software release was available. The workflow used the R analysis environment, 176 normals, min_mean_depth 200, an anchor range of 80–100, and exclude_same_gene=TRUE. CR ≥1.1 was the exploratory primary operating point; CR ≥1.5 was the native conservative comparator. Exact archived git commit: e36e6be169d4838b42d2401af373173b04dd7992.

BayesCNV was run from the public repository; no tagged software release was available. The analysis used Python 3.11.15. Target coverage was generated with mosdepth 0.3.14 and normalized to the median profile of 176 normals. The ERBB2 CNV2Hierarchical score was evaluated at μ ≥1.0, ≥1.2, and ≥1.5. Exact archived git commit: e6f247f81ff511d7049f43ec6bcc0256977a288b.

OFF-PEAK was run from the public repository; no tagged software release was available. The R analysis environment was used, and the 03_OFF-PEAK.R script was left unmodified. The workflow analyzed one 302-sample matrix comprising 126 tumors and 176 normals, with mincor 0.9, leave-one-out 1, downsample 20,000, nbFake 500, and minZ 4. The study endpoint was the native ERBB2-overlapping DUP call; no ERBB2-specific retuning was performed. Exact archived git commit: 5c2b1050797c0c4cc87fdb393f76d0fd0abbeca6.

The shared analytical environment included samtools 1.24, bedtools 2.31.1, mosdepth 0.3.14, Python 3.11.15, R 4.3.1 for the general R analysis environment, R 4.3.3 for ClinCNV, and conda 26.1.1. Software versions were recorded from the analysis environment snapshot dated 8 August 2026.

Supplementary Methods S3. No-Call Handling and Purity Stratification

Missing outputs were excluded only from the relevant configuration confusion matrix. Native PureCN produced numerical outputs for all 126 specimens; its run-level summary flags were retained separately from the numerical result (Flagged=TRUE for 126/126, Failed=FALSE for 126/126; ERBB2 C.flagged=FALSE for 126/126). PURPLE had three numerical no-calls. PURPLE status was NO_TUMOR for 102/126 specimens and was retained as a separate quality flag rather than treated as a missing numerical result.

Purity analyses were cumulative and nested at thresholds of ≥10%, ≥20%, ≥30%, ≥40%, ≥50%, ≥60%, ≥70%, ≥80%, and ≥90%. The separate strict high-purity analysis used >70%. These definitions are not interchangeable: the cumulative ≥70% stratum contained 32 tumors, whereas the strict >70% subgroup contained 30. These strata are computational and method-dependent. Independent case-linked percentage pathology cellularity was unavailable; no pathology–PureCN correlation is claimed. All subgroup analyses are exploratory.

Table S1. Sensitivity and specificity across cumulative PureCN-estimated tumor-purity strata. Each caller cell is sensitivity / specificity (%).

Purity	Cohort n	FISH+ / FISH−	CNVkit	ClinCNV	PureCN	PURPLE	CESAR	BayesCNV	OFF-PEAK
≥10%	126	59 / 67	76.3 / 91.0	67.8 / 85.1	16.9 / 95.5	58.6 / 92.3	78.0 / 82.1	84.7 / 65.7	18.6 / 98.5
≥20%	119	58 / 61	75.9 / 90.2	69.0 / 85.2	17.2 / 95.1	59.6 / 91.5	77.6 / 80.3	84.5 / 65.6	17.2 / 100.0
≥30%	107	52 / 55	75.0 / 90.9	67.3 / 85.5	17.3 / 94.5	60.8 / 92.5	76.9 / 80.0	84.6 / 65.5	15.4 / 100.0
≥40%	88	47 / 41	76.6 / 87.8	66.0 / 82.9	17.0 / 95.1	60.9 / 89.7	76.6 / 75.6	85.1 / 70.7	14.9 / 100.0
≥50%	71	38 / 33	71.1 / 87.9	63.2 / 84.8	13.2 / 93.9	62.2 / 90.3	73.7 / 72.7	84.2 / 66.7	10.5 / 100.0
≥60%	54	30 / 24	76.7 / 83.3	70.0 / 79.2	13.3 / 91.7	65.5 / 87.0	76.7 / 70.8	86.7 / 66.7	10.0 / 100.0
≥70%	32	13 / 19	92.3 / 89.5	84.6 / 84.2	15.4 / 94.7	91.7 / 89.5	100.0 / 73.7	92.3 / 73.7	7.7 / 100.0
≥80%	19	7 / 12	100.0 / 91.7	85.7 / 91.7	28.6 / 91.7	100.0 / 91.7	100.0 / 75.0	85.7 / 66.7	0.0 / 100.0
≥90%	6	2 / 4	100.0 / 100.0	100.0 / 100.0	100.0 / 100.0	100.0 / 100.0	100.0 / 75.0	100.0 / 100.0	0.0 / 100.0

Table S2. Alternative caller operating points calculated from raw per-sample calls.

Caller	Alternative rule	Evaluable	No-call	Sensitivity, %	Specificity, %	F1, %	Interpretation
CNVkit	Strict log2 ≥0.6465385	126	0	44.1	97.0	59.8	High-specificity comparator
ClinCNV	Baseline S100/L4	126	0	61.0	91.0	71.3	Baseline comparator
ClinCNV	Strict S150/L1	126	0	57.6	94.0	70.1	High-specificity comparator
PureCN	absCN ≥6	126	0	69.5	32.8	56.6	High sensitivity / low specificity
PureCN	absCN ≥8	126	0	49.2	62.7	51.3	Intermediate trade-off
PureCN	absCN ≥10	126	0	32.2	82.1	42.2	Intermediate trade-off
PureCN	absCN ≥17	126	0	13.6	100.0	23.9	Stricter than primary
PURPLE	HMF driver AMP	126	0	57.6	92.5	69.4	Native driver comparator
CESAR	Native CR ≥1.5	126	0	45.8	95.5	60.7	Conservative/native comparator
BayesCNV	μ ≥1.2	126	0	69.5	80.6	72.6	Intermediate comparator
BayesCNV	μ ≥1.5	126	0	49.2	91.0	61.7	High-specificity comparator

Table S3. Reagents, platforms, software versions, study functions, and official sources. Website sources were accessed on 20 August 2026.

Item	Version / catalog	Function in this study	Official source	Ref.
QIAamp DNA FFPE Tissue Kit	Cat. 56404	FFPE tumor DNA extraction	https://www.qiagen.com/fm/products/discovery-and-translational-research/dna-rna-purification/dna-purification/genomic-dna/qiaamp-dna-ffpe-tissue-kit	[7]
GM Blood M (Raissol)	—	Blood normal DNA extraction	https://products.sesana.ru/raissoleng	[8]
VAHTS Universal Plus DNA Library Prep Kit for MGI V2	NDM627	Enzymatic fragmentation/library preparation	https://www.vazymeglobal.com/product-center/dna-library-preparation-kit	[9]
NanOnco Plus Panel v3.0	Panel v3.0	Target enrichment / MGI capture protocol	https://www.nanodigmbio.com/Solid-Tumor/819.html	[10]
DNBSEQ-G400	—	PE150 sequencing	https://en.mgi-tech.com/Products/reagents/du_id/4/ping_id/1/category_id/	[11]
HER2/CEP17 FISH kit	FP-001	Binary FISH reference	https://healthcare-biotech.com/list_49.html	[12]
fastp	0.23.x	FASTQ QC / trimming	https://github.com/OpenGene/fastp	[13]
BWA-MEM2	2.2.1	GRCh38 read alignment	https://github.com/bwa-mem2/bwa-mem2/releases/tag/v2.2.1	[14,15]
streammd	4.3.0	Duplicate marking	https://github.com/delocalizer/streammd	[16]
Sambamba	1.0.1	BAM sorting / indexing	https://github.com/biod/sambamba	[17]
GATK	4.6.2.0	BQSR (BaseRecalibratorSpark / ApplyBQSRSpark)	https://github.com/broadinstitute/gatk/releases/tag/4.6.2.0	[18,19]
CNVkit	0.9.14	Target/antitarget depth normalization and segmentation	https://github.com/etal/cnvkit/releases/tag/v0.9.14	[23,24]
ClinCNV	v1.19.1; R 4.3.3; Python 3.11	Cohort/tumor-only CNV calling	https://github.com/imgag/ClinCNV	[25,26]
ngs-bits	2026_06	Coverage utilities supporting ClinCNV	https://github.com/imgag/ngs-bits	[27]
PureCN	2.18.0 / Bioconductor 3.23 in later snapshot; native-output run version unverified	Standalone tumor-only native absolute CN; configuration provenance caveat in Methods S2	https://github.com/lima1/PureCN	[28,29]
AMBER/COBALT/PURPLE	4.2 / 2.2 / 4.3; hmf_pipeline_resources.38_v2.3.0--2	Tumor-only allele-specific CNV stack	https://github.com/hartwigmedical/hmftools	[30,31]
CESAR	Git e36e6be169d4838b42d2401af373173b04dd7992	Exploratory ERBB2 CR calling	https://github.com/nishuai/CesaR	[32,33]
BayesCNV	Git e6f247f81ff511d7049f43ec6bcc0256977a288b	Exploratory hierarchical Bayesian CNV model	https://github.com/Pillar-Biosciences-Inc/BayesCNV	[34,35]
mosdepth	0.3.14	Target coverage for BayesCNV adaptation	https://github.com/brentp/mosdepth	[36]
OFF-PEAK	Git 5c2b1050797c0c4cc87fdb393f76d0fd0abbeca6	Native cohort-level off-target/on-target CNV call	https://github.com/mquinodo/OFF-PEAK	[37,38]
Shared analytical environment	samtools 1.24; bedtools 2.31.1; Python 3.11.15; R 4.3.1 / 4.3.3; conda 26.1.1	Shared runtime and utility versions recorded for the CNV analysis environment	https://github.com/samtools/samtools; https://github.com/arq5x/bedtools2; https://www.python.org/; https://www.r-project.org/; https://docs.conda.io/	—

Table S4. Performance in the strict high-purity subgroup (>70%;*n*=30; 13 FISH-positive and 17 FISH-negative). Sensitivity and specificity are shown with Wilson 95% confidence intervals.

Caller	Evaluable / no-call	TP / FP / FN / TN	Sensitivity, % (95% CI)	Specificity, % (95% CI)	F1, %	κ
CNVkit	30 / 0	12 / 1 / 1 / 16	92.3 (66.7–98.6)	94.1 (73.0–99.0)	92.3	0.864
ClinCNV	30 / 0	11 / 2 / 2 / 15	84.6 (57.8–95.7)	88.2 (65.7–96.7)	84.6	0.729
PureCN	30 / 0	2 / 1 / 11 / 16	15.4 (4.3–42.2)	94.1 (73.0–99.0)	25.0	0.104
PURPLE	29 / 1	11 / 1 / 1 / 16	91.7 (64.6–98.5)	94.1 (73.0–99.0)	91.7	0.858
CESAR	30 / 0	13 / 3 / 0 / 14	100.0 (77.2–100.0)	82.4 (59.0–93.8)	89.7	0.802
BayesCNV	30 / 0	12 / 4 / 1 / 13	92.3 (66.7–98.6)	76.5 (52.7–90.4)	82.8	0.670
OFF-PEAK	30 / 0	1 / 0 / 12 / 17	7.7 (1.4–33.3)	100.0 (81.6–100.0)	14.3	0.086

Table S5. Exact two-sided McNemar comparisons of caller correctness versus CNVkit on common evaluable samples. P-values are exploratory and unadjusted for multiplicity. The specimen-level tests do not adjust for repeated patient identifiers or unequal decision-rule development and are not confirmatory software comparisons.

Comparator	Common evaluable n	CNVkit correct / comparator wrong	CNVkit wrong / comparator correct	Nominal p
ClinCNV	126	11	2	0.0224609
PureCN	126	37	5	4.4337e-07
PURPLE	123	12	2	0.0129395
CESAR	126	8	3	0.226562
BayesCNV	126	22	10	0.0501025
OFF-PEAK	126	36	7	8.96304e-06

Table S6. Technical no-call summary. Sample identifiers are omitted from the publication table.

Caller	No-calls	FISH-positive no-calls	FISH-negative no-calls	Overlap note
PURPLE	3 (2.38%)	1	2	No overlap with native PureCN numerical no-calls (none observed)

Table S7. Contextual laboratory QC from a separate 36-record export (12 sample identifiers overlap the benchmark). This table is not a QC summary of the 126-specimen benchmark and cannot establish its inclusion thresholds. Case-linked benchmark coverage is supplied separately.

Parameter	Value / description
Tumor material	FFPE breast tumor sections
Tumor DNA extraction	QIAamp DNA FFPE Tissue Kit (QIAGEN)
Normal DNA extraction	GM Blood M (Raissol/Sesana)
Library preparation	VAHTS Universal Plus DNA Library Prep Kit for MGI V2 (NDM627)
Capture	Nanodigmbio NanOnco Plus Panel v3.0
Sequencer	DNBSEQ-G400
Mean target coverage, median (range)	441.8× (184.2–659.1×)
Median target coverage, median (range)	417.5× (182–631×)
Duplication rate, median	9.45%
Target bases ≥10×, median	97.15%
Post-filter Q30 rate, median	95.43%
CNVkit normal reference	167 QC-passed blood normals
PureCN NormalDB	176 blood normals
COBALT/PURPLE, CESAR, BayesCNV reference pool	176 blood normals

Table S8. Complete primary diagnostic metrics against the FISH reference. Sensitivity and specificity are shown with Wilson 95% confidence intervals. These are development-set estimates at original fixed rules, not held-out validation. The PureCN row uses corrected standalone native output with the retained, unoptimized C ≥14 threshold.

Caller	Evaluable / no-call	TP / FP / FN / TN	Sensitivity, % (95% CI)	Specificity, % (95% CI)	PPV, %	NPV, %	F1, %	Accuracy, %	κ
CNVkit	126 / 0	45 / 6 / 14 / 61	76.3 (64.0–85.3)	91.0 (81.8–95.8)	88.2	81.3	81.8	84.1	0.679
ClinCNV	126 / 0	40 / 10 / 19 / 57	67.8 (55.1–78.3)	85.1 (74.7–91.7)	80.0	75.0	73.4	77.0	0.534
PureCN	126 / 0	10 / 3 / 49 / 64	16.9 (9.5–28.5)	95.5 (87.6–98.5)	76.9	56.6	27.8	58.7	0.131
PURPLE	123 / 3	34 / 5 / 24 / 60	58.6 (45.8–70.4)	92.3 (83.2–96.7)	87.2	71.4	70.1	76.4	0.518
CESAR	126 / 0	46 / 12 / 13 / 55	78.0 (65.9–86.6)	82.1 (71.3–89.4)	79.3	80.9	78.6	80.2	0.601
BayesCNV	126 / 0	50 / 23 / 9 / 44	84.7 (73.5–91.8)	65.7 (53.7–75.9)	68.5	83.0	75.8	74.6	0.497
OFF-PEAK	126 / 0	11 / 1 / 48 / 66	18.6 (10.7–30.4)	98.5 (92.0–99.7)	91.7	57.9	31.0	61.1	0.180

Table S9. Sensitivity and specificity with Wilson 95% confidence intervals for the retained cumulative purity strata (≥20%, ≥60%, and ≥70%). Revised Figure 1 displays the full cohort, ≥60%, and ≥70%; ≥20% is retained here for continuity with the original analysis.

Purity	Caller	Cohort n	Evaluable	Sensitivity, % (95% CI)	Specificity, % (95% CI)
≥20%	CNVkit	119	119	75.9 (63.5–85.0)	90.2 (80.2–95.4)
≥20%	ClinCNV	119	119	69.0 (56.2–79.4)	85.2 (74.3–92.0)
≥20%	PureCN	119	119	17.2 (9.6–28.9)	95.1 (86.5–98.3)
≥20%	PURPLE	119	116	59.6 (46.7–71.4)	91.5 (81.6–96.3)
≥20%	CESAR	119	119	77.6 (65.3–86.4)	80.3 (68.7–88.4)
≥20%	BayesCNV	119	119	84.5 (73.1–91.6)	65.6 (53.0–76.3)
≥20%	OFF-PEAK	119	119	17.2 (9.6–28.9)	100.0 (94.1–100.0)
≥60%	CNVkit	54	54	76.7 (59.1–88.2)	83.3 (64.1–93.3)
≥60%	ClinCNV	54	54	70.0 (52.1–83.3)	79.2 (59.5–90.8)
≥60%	PureCN	54	54	13.3 (5.3–29.7)	91.7 (74.2–97.7)
≥60%	PURPLE	54	52	65.5 (47.3–80.1)	87.0 (67.9–95.5)
≥60%	CESAR	54	54	76.7 (59.1–88.2)	70.8 (50.8–85.1)
≥60%	BayesCNV	54	54	86.7 (70.3–94.7)	66.7 (46.7–82.0)
≥60%	OFF-PEAK	54	54	10.0 (3.5–25.6)	100.0 (86.2–100.0)
≥70%	CNVkit	32	32	92.3 (66.7–98.6)	89.5 (68.6–97.1)
≥70%	ClinCNV	32	32	84.6 (57.8–95.7)	84.2 (62.4–94.5)
≥70%	PureCN	32	32	15.4 (4.3–42.2)	94.7 (75.4–99.1)
≥70%	PURPLE	32	31	91.7 (64.6–98.5)	89.5 (68.6–97.1)
≥70%	CESAR	32	32	100.0 (77.2–100.0)	73.7 (51.2–88.2)
≥70%	BayesCNV	32	32	92.3 (66.7–98.6)	73.7 (51.2–88.2)
≥70%	OFF-PEAK	32	32	7.7 (1.4–33.3)	100.0 (83.2–100.0)

Supplementary Methods S4. Common Decision Calibration

The additional common decision-calibration analysis used 5-fold StratifiedGroupKFold splits repeated 20 times, with seed 20260922 plus the zero-based repeat index, grouping by recorded patient identifier. The same splits were used for all configurations. On each training set, we selected a numeric threshold maximizing sensitivity subject to specificity ≥0.90; ties favored higher specificity and then higher threshold. Threshold candidates comprised unique observed training values plus an all-negative endpoint. Scores were CNVkit median-bin log2, native PureCN absCN, PURPLE minCN/ploidy, CESAR CR, and BayesCNV μ. For ClinCNV, the same objective selected among all 28 archived scoreS×lengthS profiles at CN_change ≥2, with an all-negative fallback; tied profiles were resolved deterministically by profile name. OFF-PEAK remained a fixed native comparator because a calibrated continuous score for every specimen was unavailable. Missing numerical outputs remained no-calls. Each specimen received one held-out prediction per repeat. Point estimates were calculated from confusion-matrix contributions averaged over repeats. Percentile 95% intervals used 2,000 patient-cluster bootstrap resamples of these held-out contributions, keeping repeats together; these intervals condition on the fitted predictions and do not include complete pipeline-refitting uncertainty. Normalization, reference construction, upstream model fitting, and the original choice of score were not repeated within folds. Thus, this standardizes decision calibration on archived outputs, not end-to-end or external validation.

Supplementary Methods S5. Source Audit and Descriptive Profiles

Recovered FISH source check (45 quantitative records): the binary FISH label is retained for all 126 specimens, while quantitative HER2, CEP17, HER2/CEP17, and ISH-group fields are retained only for the 45 pathology-assessed records. The remaining 81 externally sourced records contain binary status only. Ratio-only classification agreed with the recorded binary label in 33/45 records (73.3%; Cohen κ=0.438); the operational rule combining ratio and mean HER2 copies agreed in 43/45 (95.6%; Cohen κ=0.910). The two operational discordances (ERB052 and ERB099) and one internally non-reproducible displayed ratio (ERB031) remain flagged for primary-source verification. FISH ratio is an amplification-classification variable and is not a percentage estimate of specimen tumor cellularity. No quantitative FISH or pathology-cellularity values were imputed.

Per-specimen archived scores and calls were frozen before reanalysis. Public-facing case tables use ERB001–ERB126 and patient groups P001–P123; the repeated identifier represents repeat biopsies/re-extractions from one patient and is kept together in grouped resampling. Regional profiles use only 123 CNR files whose median ERBB2 log2 agrees with the archived score to 10^{-4}. Current files for ERB057, ERB099, and ERB123 failed that compatibility check and were excluded from profile summaries. This prevents mixing revised profiles with original benchmark calls.

For multi-gene analyses, comma-separated target annotations were expanded and antitarget bins excluded. Genes required at least three bins in every compatible specimen, yielding 554 genes. For each gene/specimen, median normalized log2 and the unscaled median absolute deviation (MAD) of target-bin log2 were calculated. Between-specimen IQR and MAD summarize gene-median variation. These quantities combine biology and technical effects; no FISH labels or ERBB2 threshold were transferred to other genes. RNA expression was not measured.

Table S10. Scope of the common decision-calibration analysis.

Configuration	Input / candidates	Selection scope
CNVkit	Fixed median-bin log2; unique training values + ∞	Decision threshold only; prior feature choice remains fixed
ClinCNV	28 archived scoreS×lengthS profiles; CN_change ≥2	Select profile using training FISH only; all-negative fallback

Configuration	Input / candidates	Selection scope
PureCN	Native absCN	Decision threshold only; standalone tumor-only output
PURPLE	minCN / ploidy	Decision threshold only; quality-status limitations retained
CESAR	CR	Decision threshold only; exploratory FFPE transfer
BayesCNV	μ	Decision threshold only; exploratory FFPE transfer
OFF-PEAK	Native DUP	Fixed comparator; not retuned

Table S11. CNVkit discordance summaries at the original fixed threshold. The n column counts all specimens in each outcome. Coverage/bin-MAD summaries use only compatible CNR files: FN 14/14, FP 5/6, TN 61/61, and TP 43/45. Case-linked quantitative FISH fields were recovered for 45 of 126 specimens by our pathology team; the remaining 81 specimens had binary FISH status from an external collaborating organization without quantitative signal fields. Independent percentage pathology cellularity was not recovered. Quantitative-FISH fields were not used for the full-cohort performance tables, and these specimen-level descriptions are exploratory.

Outcome	n	Median log2	Median computational purity	Median ERBB2 bin depth, ×	Median bin log2 MAD
FN	14	-0.037	60.5%	494.3	0.168
FP	6	0.555	67.5%	706.0	0.205
TN	61	-0.061	47.0%	497.5	0.182
TP	45	0.751	60.0%	1073.6	0.185

Table S12. Descriptive variation for selected panel genes in 123 specimens with compatible CNR files. Complete summaries for all 554 eligible genes are supplied in multigene_signal_variability.tsv. Units are normalized DNA log2 ratios. These are not estimates of diagnostic accuracy or technical variance in isolation.

Gene	Min. bins	Median gene log2	Between-specimen IQR	Between-specimen MAD	Median within-gene bin MAD
CCND1	5	0.054	0.507	0.274	0.176
EGFR	59	-0.035	0.267	0.134	0.236
ERBB2	28	0.064	0.518	0.172	0.185
FGFR1	48	-0.109	0.364	0.167	0.200
MDM2	12	0.064	0.240	0.135	0.156
MET	108	0.224	0.244	0.128	0.169
MYC	10	0.611	0.560	0.283	0.184

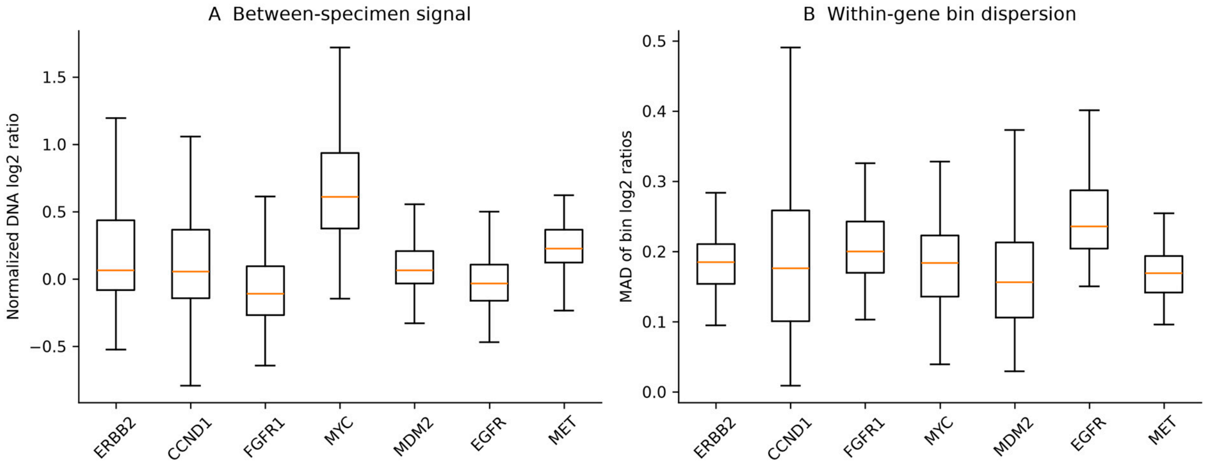

Figure S1. Descriptive normalized DNA-depth variation for selected panel genes. (A) Distribution of specimen-level median log2; (B) distribution of within-gene bin-log2 MAD. Boxes show median and interquartile range; whiskers extend to 1.5× IQR and outliers are hidden for readability. Gene-specific biological alterations and technical variability both contribute. No non-ERBB2 diagnostic validation is implied.

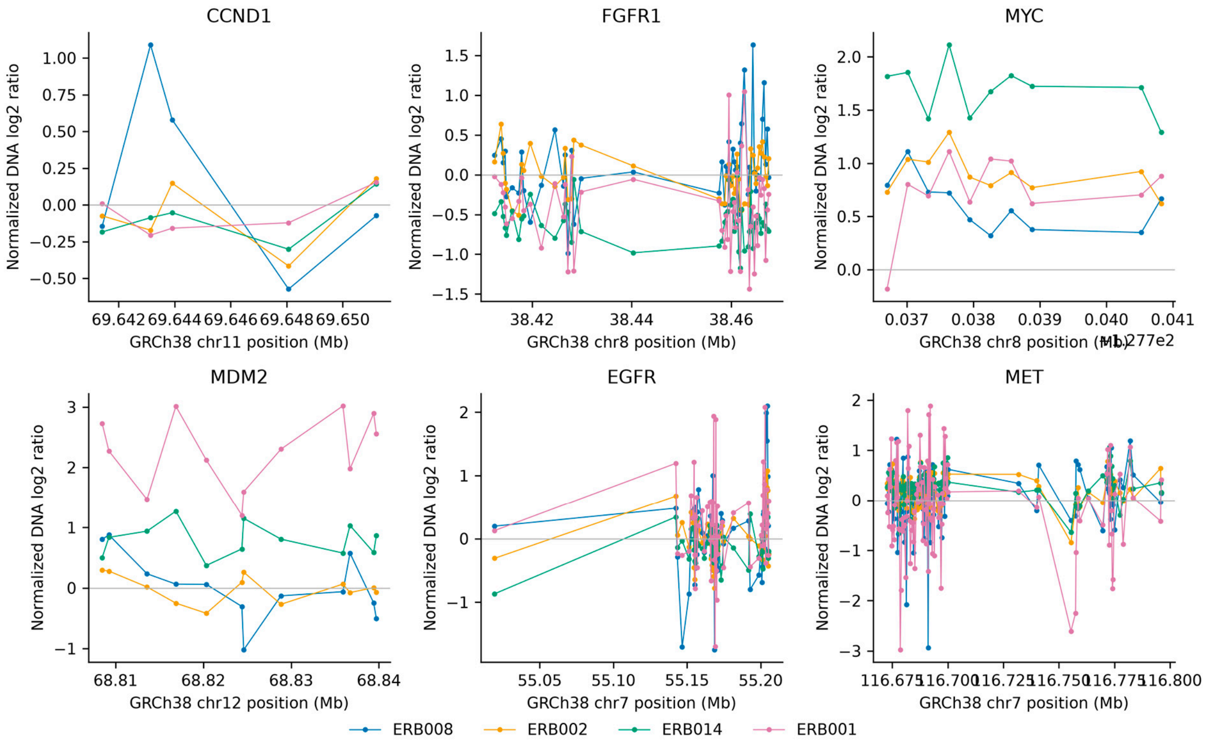

Figure S2. Target-bin DNA-depth profiles at six other panel genes for the same four deterministic cases used in Figure 3. Lines connect measured target bins and do not imply continuous coverage between targets. Positions use GRCh38. These observations provide regional signal context only; no gene-specific threshold or experimental validation is implied.

Supplementary Data S1. all_cases.tsv and discordant_cases.tsv provide pseudonymous identifiers, binary FISH, computational purity, available QC, continuous outputs, original calls, and discordance classes. Quantitative FISH fields are present for the 45-case pathology-assessed subset; unavailable quantitative FISH and pathology fields are explicitly NA for the remaining records, including the 81 externally sourced binary-status records. patient_group allows repeated specimens to remain together in resampling. Decision thresholds are specified in main Table 1. cnvkit_score_log2 is the rounded historical export;

cnvkit_score_log2_full_precision is the value to use for exact threshold comparisons, including the boundary specimen ERB039. Supplementary Data S2. cv_splits.tsv, training_selections.tsv, oof_predictions.tsv, oof_metrics.tsv, and oof_metrics_by_repeat.tsv document every calibration split and held-out prediction. Supplementary Code. analyze.py and pinned dependencies reproduce these tables and figures from the separately supplied input dataset. The local code package contains only caller-level analysis and excludes alignment and duplicate-marking workflows; public deposition is pending.

References

Reference numbers in the Supplementary Materials correspond to the numbered reference list in the main manuscript.
